# Sacrifice of Involved Nerve Root during Surgical Resection of Foraminal and/or Dumbbell Spinal Neurinomas

**DOI:** 10.3390/brainsci13010109

**Published:** 2023-01-07

**Authors:** Alberto Vandenbulcke, Ginevra Federica D’Onofrio, Gabriele Capo, Wassim Baassiri, Cédric Y. Barrey

**Affiliations:** 1 Department of Spine and Spinal Cord Surgery, Hôpital Pierre Wertheimer, Hospices Civils de Lyon, Claude Bernard University of Lyon 1, 59 Boulevard Pinel, 696777 Lyon-Bron, France; 2 Department of Neurosurgery, University Hospital of Lausanne, 46 Rue du Bugnon, 1011 Lausanne, Switzerland; 3 Department of Neurosurgery, Agostino Gemelli Hospital, Via Pineta Sacchetti 217, 00168 Rome, Italy; 4 Department of Neurosurgery, IRCCS Humanitas Research Hospital, Via Manzoni 56, 20089 Milan, Italy; 5 Laboratory of Biomechanics, ENSAM, Arts et Metiers ParisTech, 153 Boulevard de l’Hôpital, 75013 Paris, France

**Keywords:** spinal nerve sheath tumors, foraminal schwannoma, spinal cord, spinal surgery, intradural lesion, nerve root sacrifice

## Abstract

Even if usually needed to achieve the gross total resection (GTR) of spinal benign nerve sheath tumors (NSTs), nerve root sacrifice remains controversial regarding the risk of neurological deficit. For foraminal NSTs, we hypothesize that the involved root is poorly functional and thus can be safely sacrificed. All spinal benign NSTs with foraminal extension that underwent surgery from 2013 to 2021 were reviewed. The impacts of preoperative clinical status and patient and tumor characteristics on long-term outcomes were analyzed. Twenty-six patients were included, with a mean follow-up (FU) of 22.4 months. Functional motor roots (C5-T1, L3-S1) were involved in 14 cases. The involved nerve root was routinely sacrificed during surgery and GTR was obtained in 84.6% of cases. In the functional root subgroup, for patients with a pre-existing deficit (n = 5/14), neurological aggravation persisted in one case at last FU (n = 1/5), whereas for those with no preop deficit (n = 9/14), a postoperative deficit persisted in one patient only (n = 1/9). Preoperative radicular pain was the only characteristic significantly associated with an immediate postoperative motor deficit (*p* = 0.03). The sacrifice of an involved nerve root in foraminal NSTs seems to represent a reasonable and relevant option to resect these tumors, permitting one to achieve tumor resection in an oncologic fashion with a high rate of GTR.

## 1. Introduction

Spinal nerve sheath tumors (NSTs) are common intradural spinal tumors [1,2], usually arising from the dorsal sensory roots [3]. They present a ubiquitarian localization in the spine, although a more common incidence in the cervical and lumbar tracts is reported [4,5,6,7]. The majority of these lesions (60%) are intradural extramedullary, 25% are purely extradural and a further 15% have both intradural and extradural components. Less than 1% are intramedullary [3]. In 15% of cases, they extend laterally through the nerve root origin, assuming a dumbbell shape [8], representing the most common spinal foraminal lesion. Local progression may lead to spinal cord compression, bony erosion and subsequent deformity.

NSTs include schwannomas, neurofibromas and malignant nerve sheath tumors (MNST). Spinal schwannomas originate from the Schwann cells, with an eccentric growth pattern [9], whereas neurofibromas are mostly peripheral NSTs arising from the endoneurium and encasing the nerve root [10]. Whenever they occur in the spine, both are benign and have a prevalence for transdural and foraminal extension in 15% of cases [4]. MNST are rare yet aggressive soft tissue sarcomas of neural origin that may occasionally occur in the spine. Prognosis is poor, with a high rate of relapse and mortality (between 23% and 69%) [11]. In order to obtain total resection with free margins, root sacrifice is routinely performed.

The gold-standard treatment for benign NSTs (WHO grade I schwannomas and neurofibromas) is radical resection with neurological function preservation. Recurrence occurs in up to one third of cases at 15 years [12]. In contrast to pure intradural lumbar schwannoma, entire nerve root sacrifice for intra-foraminal and/or dumbbell NSTs is controversial regarding the risk of permanent motor dysfunction and neuropathic pain [7,13,14,15]. Even if histological studies have shown that the parent nerve progressively loses its function [16], and several surgical series have reported a low incidence of permanent postoperative neurological deficits after root sacrifice [17,18,19,20], surgeons often opt for root preservation. Consequently, foraminal extension is associated with a higher risk of subtotal resection (STR) [7,20]. In contrast to peripheral nerve tumors, we assume that foraminal/dumbbell tumors lead to the chronic compression of the root against the stiff bony limits of the foramen, with progressive loss of nerve function. We therefore hypothesize that most of these involved roots are non- or poorly functional, with compensation by adjacent roots cranially and caudally, and could thus be sacrificed with no or very limited postoperative deficit. The sacrifice of the involved nerve root presents many advantages, permitting one to remove the tumor in total, in an “en bloc” fashion, making the surgery easier, faster, less hemorrhagic and more respectful of the principles of oncologic resection, thus reducing the risk of tumor recurrence [21]. Nowadays, little is known about the predictive risk factors of postoperative deficit with root sacrificed [13].

The aim of this study is therefore to report the neurological outcomes in a retrospective series of spinal dumbbell and intraforaminal benign NSTs treated with systematic nerve root amputation and to evaluate the predictive factors of postoperative deficit.

## 2. Materials and Methods

### 2.1. Study Design

We conducted a retrospective analysis of all the patients treated for spinal benign NSTs (WHO grade I schwannomas and neurofibromas) with foraminal extension (pure foraminal and/or dumbbell lesions), between 2013 and 2021, at the Spinal Department of the Neurological Hospital in Lyon (France). Complete resection of the parent spinal nerve was performed systematically. Patients with a minimum follow-up of 6 months, and with pre- and postop contrast-enhanced magnetic resonance imaging (MRI), were included. Demographic, clinical, radiological and operative data were collected and analyzed. Functional roots, from C5 to T1 and L3 to S1, were considered as a subgroup. Presenting signs and symptoms were classified as axial pain (cervical, lumbar and dorsal), radicular pain (RP) (if neuropathic pain and/or sensory disturbance related to the affected nerve root occurred), radicular motor deficit (MD) and sensory loss (SL) and myelopathy (pyramidal or sensory tracts signs and symptoms). Strength was reported according to the Medical Research Council (MRC) scale. Motor deficit was defined as severe and moderate if MRC ≤2 and ≥3, respectively. In the first postoperative day, any reduction in sensory and motor function into a dermatome or myotome compatible with the sacrificed spinal nerve was considered as an immediate postoperative radicular deficit (RD). Any radicular deficit was considered persistent if it did not yield to a preoperative level at 12 months. The evolution of pre-existing neuropathic pain and new onset were also recorded. Tumor location and extension, foraminal enlargement and degree of resection (GTR and STR) were assessed on the pre- and postoperative MRI. Tumors were classified according to the Sridhar classification [22]. Only classes III to V (foraminal extension) were included in the present study.

Institutional review board approval was obtained before conducting the study (CSE-HCL protocol number 22_5916).

### 2.2. Statistical Analysis

Parametric data were expressed as means ± standard deviation and compared via the Student *t*-test. For categorical variables, Fisher’s exact test was performed. Significance was assessed at *p* < 0.05. Univariate analysis was performed to study the correlation between postoperative deficit and tumor location and extension, foraminal enlargement, preoperative signs of myelopathy, radicular pain and MD and or SL, patient’s sex, age, histologic type, extent of resection and symptom duration. Univariate analysis for sensory and/or motor radicular deficit was performed for the whole series, while only the motor deficit was analyzed for the functional root subgroup. Statistical analysis was performed using the statistical software package STATA version 17 (College Station, TX, USA, StataCorp LP).

## 3. Results

### 3.1. Patient Population

Clinical and demographic data are reported in Table 1. We selected 26 patients operated for a benign NST with foraminal extension at our institution between 2013 and 2021. The parent nerve root was completely resected in all cases and the tumor resected in an “en bloc” fashion in the majority of cases. Functional nerve roots were involved in 14 cases (53.8%), five in the upper (C5 to T1) and nine in the lower (L3 to S1) spine.

Mean patient age was 47 years (range 22–76). All procedures were primary resections, except one patient who had prior surgical treatment 4 years earlier with a partial tumor resection without neurological sequelae. The most frequently presenting symptom was axial pain in 18 cases (69%). RP was reported in 11 patients (42%). In the functional nerve root subgroup, five patients (35.7%) had a radicular motor deficit preoperatively, classified as moderate in all cases (MRC ≥ 3). Ten patients had myelopathic signs and/or symptoms. Concerning localization, tumors were predominant in the lumbosacral region (n = 13, 50%), followed by the cervical (n = 10, 38.5%) and thoracic (n = 3, 11.5%) regions. Almost half of the patients (n = 12, 46.2%) presented a Sridhar type IV tumor, n = 8 (30.8%) a type III and n = 6 (23%) a type V (Table 1). In 10 cases, the lesions extended along two or more vertebral body, and in four cases they involved two adjacent foramens. The average lesion transverse and anteroposterior diameters were 45.4 mm (range 12–80 mm) and 34.5 mm (range 11–60 mm), respectively, and in 20 (77%) cases caused enlargement of the intervertebral foramen (i.e., foraminal scalloping).

### 3.2. Surgical Procedures and Tumor Recurrence

Surgical data are reported in Table 2. Intraoperative neurophysiological monitoring (IONM) was used in all cases. Motor-evoked potential recordings were stable in amplitude in all cases during surgery. Most patients (n = 22, 84.6%) underwent single-stage surgery with a posterior approach. In four (15.4%) cases, two-stage surgery was performed to obtain complete resection. Three patients underwent posterior instrumentation with fusion at the same stage.

**Figure 1:**
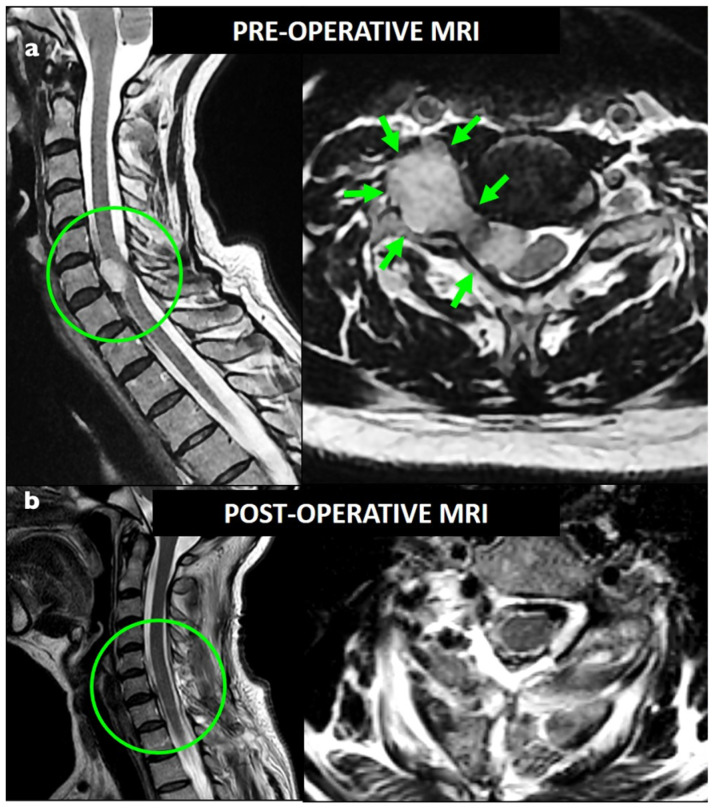
Preoperative (**a**) and postoperative (**b**) T2-weighted MRI showing a foraminal C7 dumbbell schwannoma (Sridhar class IVa); GTR was obtained with a single-stage posterior approach.

**Figure 2:**
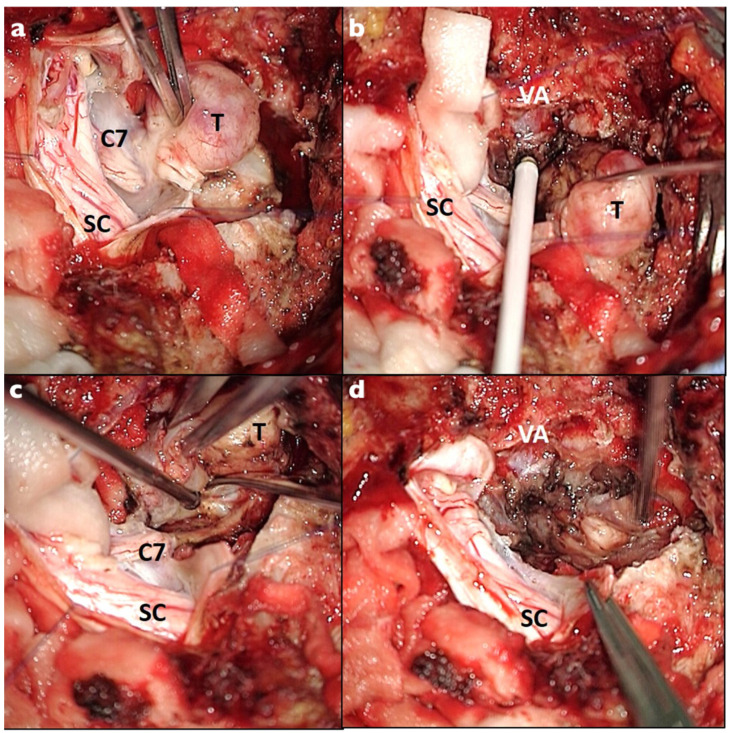
Intraoperative photos of the C7 foraminal dumbbell schwannoma. A posterior midline approach and two-level laminectomy were performed: (**a**) the lesion was dissected from the spinal cord (SC); the C7 root was identified and completely sacrificed; (**b**) the lesion was mobilized thanks to the nerve resection and the vertebral artery (VA) was identified using the intraoperative doppler; (**c**,**d**) dissection of the extraforaminal part and “en bloc” resection of the lesion was performed.

Careful dissection of the surrounding nerve rootlets and roots was performed in order to identify and sacrifice exclusively the parent spinal root. Once the parent nerve root was identified, complete resection was performed at the most distal and proximal point at the junction between the lesion and the nerve (Figure 1 and Figure 2). The intracanal and foraminal part was successfully resected in all cases. GTR resection was achieved in 22 patients (84.6%) and was obtained with a complete en bloc resection in 18 cases (69.2%). Four patients (15.4%) with a huge paraspinal component (3 Sridhar grade IVB and 1 grade V) had STR. The residual part was paravertebral in all cases. No second-stage surgery was considered necessary in these cases according to the clinical context, and no progression was observed at follow-up. Surgical complications occurred in four (15.4%) patients: two CSF leaks and two wound infections. All required surgical management. The mean postoperative follow-up was 22.4 months (range 6–85 months). No recurrence was observed.

### 3.3. Neurological Outcomes of the Entire Cohort

At the last follow-up, seven patients (26.9%) with preoperative radicular pain completely recovered; six patients (23.1%) still suffered from RP and, among them, two (7.7%) had new postoperative RP onset (Table 3).

**Figure 3:**
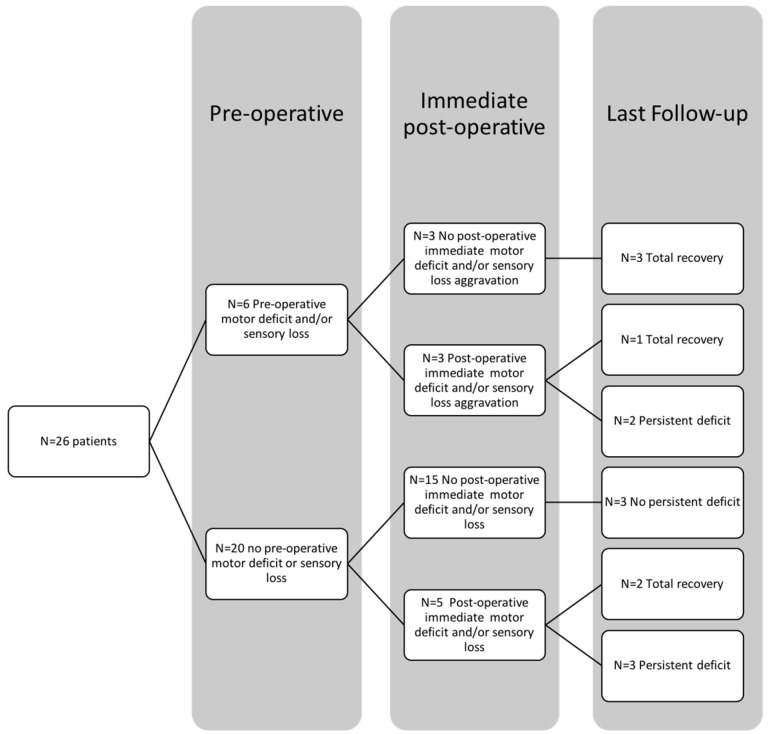
Diagram showing radicular deficit evolution in the entire cohort, from preoperative status to last follow-up evaluation.

Postoperative RD occurred in eight patients (30.8%). Four of them recovered completely, while five (19.2%), improved partially and had a mild persistent deficit (sensory and/or motor) at last FU (Table 3). For patients with a pre-existing deficit (n = 6/26, i.e., 23.1%), neurological aggravation was observed in three cases (n = 3/6, i.e., 50%) and persisted in two cases at last FU (n = 2/6, i.e., 33%), whereas for patients with no preop deficit (n = 20/26, i.e., 76.9%), a postoperative deficit was found in five patients (n = 5/20, i.e., 25%) and finally persisted in three patients (n = 3/20, i.e., 15%). (Figure 3). All the patients with permanent postoperative RD presented with radicular pain preoperatively (*p* = 0.007).

Temporary myelopathy symptoms occurred postoperatively in two cases (7.7%). At the last follow-up, six patients (n = 6/10, 60%) with sign(s) of preoperative myelopathy recovered completely and four patients (n = 4/10, 40%) improved partially. No worsening was observed.

### 3.4. Motor Outcome for Functional Roots

**Figure 4:**
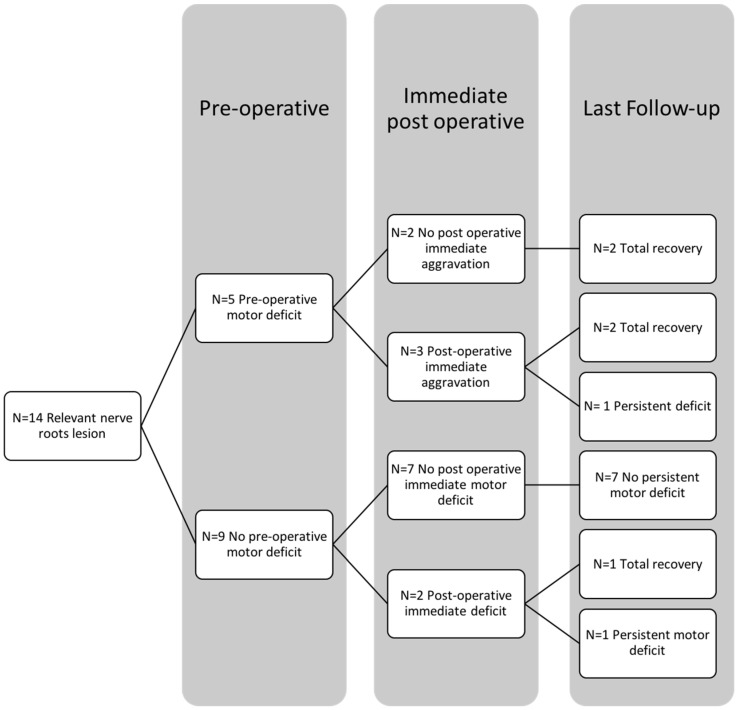
Diagram showing motor deficit evolution in the functional nerve root group, from preoperative status to last follow-up evaluation.

Postoperative MD occurred in five cases (n = 5/14, 35.7%) (Table 4); one had severe dysfunction (MRC = 1). At the last follow-up, three patients recovered completely, and the last two (14.2%) with a persistent deficit improved to MRC 4+ and 3, respectively. In this group, for patients with a pre-existing deficit (n = 5/14, i.e., 35.7%), neurological aggravation was observed in three cases (n = 3/5, i.e., 60%) and persisted in one case at last FU (n = 1/5, i.e., 20%), whereas for patients with no preop deficit (n = 9/14, i.e., 64.3%), a postoperative deficit was found in two patients (n = 2/9, i.e., 22.2%) and finally persisted in one patient (n = 1/9, i.e., 11.1%) (Figure 4). Compared to the preoperative status, MD prevalence decreased by more than half (35.7% vs. 14.2%) (*p* = 0.38) (Table 4), and dysfunction was never severe (MRC ≥ 3). All patients with immediate (*p* = 0.03) and persistent (*p* = 0.47) postoperative MD had signs of RP preoperatively.

In five cases, preoperative EMG was performed. All three patients with signs of parent nerve root sufferance had immediate postoperative MD (*p* = 0.1), and in one case, it was persistent.

### 3.5. Histopathology

Histological examination revealed a schwannoma (WHO grade I) in 24 cases (92.3%) and neurofibroma (WHO grade I) in two (7.7%) cases.

### 3.6. Risk Factor Analysis

At univariate analysis, the presence of preoperative RP was the only factor that significantly predicted immediate (*p* = 0.03) and persistent (*p* = 0.007) postoperative RD in the entire cohort (Table 5). Moreover, preoperative RP was associated with immediate postoperative MD in the functional root subgroup (*p* = 0.03) (Table 6). Analyzing the characteristics of patients who developed a postoperative deficit, they were older, with newly symptomatic, large lesions (Table 5 and Table 6).

In the entire cohort, the presence of preoperative MD and/or SL was shown to double the risk of postoperative immediate (50% vs. 25%, *p* = 0.33) and persistent (33% vs. 15%, *p* = 0.56) RD compared to asymptomatic patients. Moreover, in patients with persistent RD, the lesions had major extraforaminal and paravertebral extension (mean transverse diameter 58.6 ± 15.3 mm versus 41.8 ± 19.2 mm, *p* = 0.08). Finally, immediate and persistent RD was observed in 50% (*p* = 0.06) of patients with Sridhar grade V tumors, and no cases of complete recovery were observed in this subgroup (Table 5).

In the functional root subgroup, all the patients with persistent postoperative MD had newly onset radicular symptoms (mean medical history for persistent MD: 2 ± 0 months vs. 27.8 ± 23.4 months, *p* = 0.15). Postoperative immediate and persistent MD occurred most commonly in patients with pre-existing MD (60% versus 22% and 20% versus 11%, respectively). Moreover, patients with persistent MD had major paravertebral extension (mean transverse diameter 55.5 ± 10.6 mm vs. 41.9 ± 20.9 mm, *p* = 0.39) and the difference increased for the immediate deficit (58.6 ± 9.3 mm vs. 38.6 ± 20.2 mm, *p* = 0.06). As for the entire cohort, patients with grade V lesions developed immediate and persistent postoperative MD in 50% of cases (*p* = 0.06) (Table 6).

## 4. Discussion

Even if still controversial, amputation of a single rootlet is widely performed for intradural NSTs [7,12,13,14,15,16,17,19,23,24]. Immediate postoperative deficit is reported in 0% to 55% of cases for functional roots [13,14,19,23] and in 0% to 20% of cases for all the spinal levels [7,12,15,16,17,19]. In almost all cases, an improvement or complete recovery is observed at follow-up and it is rarely debilitating [13,23]. Moreover, complete nerve amputation is routinely performed in MNST surgery in order to obtain complete en bloc resection with free margins. Sacrifice of the parent nerve in benign foraminal dumbbell NSTs is less accepted, although no significant increase in long-term postoperative deficit was reported previously [13,14,15,18]. In our series, the appearance of a new motor deficit was observed in 35.7% of cases and a mild deficit persisted in 14.2%.

Intradural NSTs cause a gradual loss of function of the affected rootlets [13,14,16,18,19], and it is compensated by the concomitant reinnervation of the dependent peripheral structures via the nerve endings of the roots [23]. The same results can be expected for lesions extending through the neural foramen. Rather, compensation may be enhanced by progressive and chronic compression against the stiff bony limits of the neural foramen [13]. Reinnervation of the relevant spinal nerve is especially facilitated in the cervical and lumbar plexus, which provide a rich anastomotic network [13,23]. Consequently, invasiveness of the spinal plexus may compromise functional outcomes.

Foraminal NSTs, compared to pure intradural lesions, tightly encase the entire spinal nerve, increasing root deformation and adhesion. This makes root preservation challenging, and sometimes more deleterious than sacrifice (longer operation time, increased blood loss and damage to spinal cord or to other rootlets/roots, etc.). Moreover, postoperative MD was associated with nerve sacrifice in only 50% of cases by Safaee et al. [7]. Contrarily, peripheral NSTs do not have a distal anatomic network and nerve amputation is associated with a high risk of neurological impairment; therefore, enucleation is suggested.

In our series of 26 dumbbell NSTs, a global improvement in the neurological status was observed following complete nerve root amputation. We are not aware of previous larger series focused on this localization. As with Butenschoen et al. [19], we did not observe a severe motor deficit at last follow up. In most of the patients, nerve sacrifice did not produce persistent MD and 80% of the patients with a preoperative deficit completely recovered postoperatively. All the patients (n = 5) who experienced new, immediate postoperative MD improved at FU. Three patients completely recovered, and in the last two (14.2%), MD improved to MRC ≥ 3. The immediate and persistent MD were an aggravation of pre-existing MD in 60% and 20% of cases, respectively (Figure 3). We observed a high rate of new postoperative and pre-existing MD recovery accordingly with the current literature. Recovery of pre-existing and new postoperative deficits probably follows the same pathophysiologic mechanism [13,18,23]. We hypothesize that the functional recovery and cross-innervation by adjacent nerves may be enhanced by the combination of nerve deafferentation and intensive physical therapy. Among the entire cohort, we analyzed the MD, SL and RP evolution. SL is frequently underappreciated. It may be disabling, especially if concomitant MD occurs at the extremities, and should not be neglected. Concerning the RP, at last follow-up, its prevalence consistently decreased compared to the preoperative status (42% vs. 23%). Among the six patients with persistent RP, four already suffered preoperatively, and in only two cases, neuropathic pain occurred following parent nerve sacrifice. In no cases was postoperative pain debilitating and it was always controlled by a step I WHO analgesic ladder. Finally, nerve root sacrifice does not seem to improve the risk of postoperative RP compared to previous series [25].

We performed a univariate analysis of the entire cohort to identify the risk factors for postoperative RD and MD. Previous studies suggested the cervical location, histology, neurofibromatosis and symptomatic NSTs as negative predictive factors for intradural NSTs [12,13,14,19], but their role was not confirmed in foraminal lesions [18]. Normal clinical findings were previously reported as a sign of complete functional compensation (of the affected nerve) by the adjacent spinal nerve, while radicular symptoms represented nerve sufferance without complete function resumption [12,14,15,19]. In our series, patients with persistent RD and MD tended to be older, with recent onset of RP and/or deficit. Moreover, they presented larger lesions, with major extraforaminal and paravertebral extension. Preoperative RP was the only factor significantly associated with immediate and persistent postoperative deficits in the entire cohort. Furthermore, subgroup analysis for functional nerve roots showed that all the patients with immediate and persistent postoperative motor deficits presented with preoperative radicular pain. Preoperative motor and/or sensory deficits seem to increase the risk of postoperative RD and MD (Table 5 and Table 6), although no significant difference was observed, probably because of the small sample size. RP, and not preoperative motor and/or sensory deficit, was revealed to be significantly predictive of a persistent deficit following nerve sacrifice. RP could be a more reliable and sensitive sign of nerve sufferance, without complete compensation by the surrounding nerves and residual functionality of the root [12,14,15,19]. Theoretically, a protracted medical history may indicate a higher chance of functional compensation. However, symptom duration, as a predictive factor, was not previously established. In our series, we found that patients with persistent postoperative MD had a more recent medical history compared to patients without a deficit (2 ± 0 months vs. 27.8 ± 23.4 months), even if the difference was not significant (*p* = 0.15).

The data in our study suggested that paravertebral extension may affect the adjacent nerve roots and compromise compensation, especially in the lumbar spine with extension to the lumbar plexus. All persistent sensory and motor postoperative deficits occurred in lesions with paraspinal or vertebral body invasion, Sridhar class IV and V lesions, in our series. Similar results were reported by Butenschoen et al. [18]. Moreover, patients who developed RD and MD both had larger lateral extension compared to untouched patients (Table 5 and Table 6). Finally, lesions with extension through multiple foramens showed a higher rate of persistent postoperative deficit compared to mono-foraminal NSTs in the entire cohort (50% vs. 14%, *p* = 0.15) and in the functional root subgroup (33% vs. 9%, *p* = 0.39). We hypothesize that the postoperative deficit may consequently be due to a combination of the compromised compensation via the compression of adjacent roots and the dissection of the paravertebral extension of the tumor, and not related to the sacrifice of the parent nerve root. Indeed, adjacent peripheric fibers adherent to the paravertebral portion are at risk of being accidentally sacrificed during the resection. We suggest that special attention is paid to these lesions, which may be considered more similar to peripheral NSTs.

Preoperative signs of denervation on the EMG are associated with a higher risk of postoperative deficit in previous reports [12,13,14,17]. In our study, only five patients had preoperative EMG. All the patients with signs of EMG denervation had at least a temporary postoperative RD (*p* = 0.1), but this did not reach significance.

To determine parent nerve root sacrifice, intraoperative triggered electromyogram (tEMG) has been proposed to identify residual function [5]. tEMG has high specificity (94.7%) and safe resection may be performed with a significant (80%) amplitude reduction [5,26]. However, the sensitivity is still low (37.5%) and it tends to overestimate residual motor function and detect subclinical responses. Safe resection occurred despite positive responses [26]. tEMG is a useful intraoperative tool to detect residual functions, but due to its low sensitivity and the positive functional outcome reported with systematic nerve amputation, it should not preclude nerve sacrifice.

Concerning the GTR, the extension in multiple anatomical compartments, intradural, intraforaminal and paraspinal, may be technically challenging. It is significantly associated with a higher risk of STR in the literature [7,12]. The recurrence rate is not negligible and it occurred in up to 30% of spinal NSTs at 15 years [12]. The risk of recurrence has been reported to be increased by four times in patients who undergo intralesional resection compared to en bloc removal [21]. Moreover, it may be even higher in patients affected by neurofibromatosis and in MNST [4]. GTR in dumbbell NSTs without nerve root sacrifice is achieved in 22% to 60%, while nerve root sacrifice increases the GTR rate up to 96% [12,18]. In our series, GTR was obtained in 84.6% of cases. In all the cases of STR, the intracanal and intraforaminal parts were completely resected and residue remained in the paraspinal region, and patients were stable at follow-up. No cases of recurrence were observed.

Different histopathologic entities were not significantly associated with postoperative deficit. Spinal neurofibromas are generally associated with a lower incidence of postoperative deficit [13,14]. A diffuse growth pattern compared to eccentric schwannomas is supposed to rapidly compromise nerve function and decrease the risk of postoperative deficit. In our series, we reported only two cases of neurofibroma and none of them developed a persistent motor deficit. Due to the rarity of these lesions, less is known about dumbbell neurofibromas, and no definitive evidence is available.

## 5. Limitations

The main limitations of this study are the retrospective design, limited follow-up and the absence of functional scores to better quantify the impact of RD on quality of life. Moreover, dumbbell NSTs are rare lesions and our series included only 26 patients. The small sample size may have compromised the statistical analysis and the results of the univariate analysis should be interpreted cautiously. The comparison of pre- and postoperative EMG may show interesting results for the risk factor analysis, but postoperative EMG was not performed because it would not change the patient’s management.

## 6. Conclusions

Our findings support the notion that systematic nerve root sacrifice during foraminal/dumbbell NST surgery does not result in severe, persistent MD and represents a relevant option for spine surgeons during resection of these tumors. In the series, complete nerve root amputation was associated with a general improvement in the neurological status and with a high rate of GTR. It seems a valuable adjunct to resect otherwise incompletely resectable lesions without a significant increase in morbidity and permitted us to achieve tumor resection in a more “oncologic” fashion. The dysfunction was mild and root sacrifice was never debilitating.

Clinical and electrophysiological signs of nerve sufferance showed a tendency to be predicting factors of postoperative deficit, while the role of paraspinal extension should be clarified. In our practice, preoperative RP and/or deficit do not contraindicate nerve root sacrifice but are integrated into the preoperative interview and used for the interpretation of immediate MD. Prospective comparative studies are necessary to confirm these results.

## Figures and Tables

**Table 1:** Demographical, clinical and radiological characteristics of the cohort at baseline.

Characteristics	Total Population, n = 26
Age, mean ± SD, years	47 ± 16
Sex, male, n (%)	14 (54)
Location, n (%)	
Cervical	10 (38.5)
Thoracic	3 (11.5)
Lumbar	13 (50)
Side, n (%)	
Left	12 (46.2)
Right	14 (53.8)
Size, mean ± SD, mm	
Transverse	45.4 ± 19.1
Antero-posterior	34.5 ± 12.8
Foraminal enlargement, n (%)	20 (77)
Extension in multiple foramen, n (%)	4 (15.4)
Symptom duration mean ± SD, months	21 ± 19.9
Clinical presentation	
Radicular pain, n (%)	11 (42)
Motor deficit, n (%) *	5 (35.7)
Sensory loss, n (%)	1 (3.8)
Axial pain, n (%)	18 (69)
Medullary, n (%)	10 (38.5)
Motor, n (%)	8 (30.8)
Sensory, n (%)	10 (38.5)
Sphincteric, n (%)	1 (3.8)
C5-T1, n (%) *	5 (35.7)
L3-S1, n (%) *	8 (64.3)
Sridhar classification	
I, II (intraspinal)	0 (0)
III (foraminal extension)	8 (30.8)
IVA (dumbbell with extraspinal extension <2.5 cm)	4 (15.4)
IVB (dumbbell with extraspinal extension ≥2.5 cm)	8 (30.8)
V (giant erosive tumor)	6 (23)

SD standard deviation, * among N: 14 patients.

**Table 2:** Surgical characteristics of the cohort.

Characteristics	Total Population, n = 26
GTR, n (%)	22 (84.6)
STR, n (%)	4 (15.4)
Surgical approach	
Posterior, n (%)	22 (84.6)
Combined, n (%)	4 (15.4)
Instrumentation, n (%)	3 (11.5)
Surgical complications other than neurological	
CSF leak, n (%)	2 (7.7)
Wound infection, n (%)	2 (7.7)
Others	0 (0)
Follow-up, mean ± SD (min-max), months	22.4 ± 20.3 (6–85)

GTR gross total resection, STR subtotal resection, CSF cerebrospinal fluid, SD standard deviation.

**Table 3:** Reported postoperative functional outcomes of the entire cohort.

Characteristics	Total Population, n = 26
Permanent postoperative radicular pain, n (%)	6 (23)
Immediate postoperative radicular deficit #, n (%)	8 (30.8)
Motor, n (%)	1 (3.8)
Sensory, n (%)	3 (11.5)
Motor and sensory, n (%)	4 (15.4)
Permanent postoperative radicular deficit, n (%)	5 (19.2)
Motor, n (%)	2 (7.7)
Sensory, n (%)	3 (11.5)
Motor and sensory, n (%)	0 (0)
Temporary postoperative myelopathy, n (%)	2 (7.7)
Preoperative myelopathy evolution, n (%)	10 (40)
Recovery *, n (%)	6 (60)
Improvements *, n (%)	4 (40)

# including new deficit or aggravation of preoperative deficit, * among n= 10 patients.

**Table 4:** Postoperative neurological outcomes of the functional nerve roots.

Characteristics	Population, n = 14
Preoperative radicular pain, n (%)	8 (57.1)
Preoperative motor deficit, n (%)	5 (35.7)
Immediate postoperative motor deficit #, n (%)	5 (35.7)
Persistent postoperative motor deficit #, n (%)	2 (14.2)

# including new deficit or aggravation of preoperative deficit.

**Table 5:** Correlation between baseline characteristics and postoperative immediate and persistent radicular deficit in the entire cohort.

Risk Factors/Variables	Population	Immediate Radicular Deficit	*p* Value	Persistent Radicular Deficit	*p* Value
Clinical presentation					
No preoperative motor deficit and/or sensory loss, n (%)	20	5 (25)		3 (15)	
Preoperative motor deficit and/or sensory loss, n (%)	6	3 (50)	0.33	2 (33)	0.56
No preoperative radicular pain, n (%)	15	2 (13)		0 (0)	
Preoperative radicular pain, n (%)	11	6 (55)	**0.03**	5 (45)	**0.007**
No preoperative myelopathy, n (%)	16	6 (37.5)		4 (25)	
Preoperative myelopathy, n (%)	10	2 (20)	0.40	1 (10)	0.61
Medical history, mean ± SD, years	22.7 ± 15.0 *	17.6 ± 23.3	0.50		
Medical history, mean ± SD, years	22.1 ± 16.7 **			17.54 ± 30.5	0.63
No foraminal enlargement, n (%)	6	1 (17)		1 (17)	
Foraminal enlargement, n (%)	20	7 (35)	0.63	4 (20)	1
No extension in multiple foramen, n (%)	22	6 (27)		3 (14)	
Extension in multiple foramen, n (%)	4	2 (50)	0.56	2 (50)	0.15
Location					
Cervical, n (%)	10	3 (30)	1	1 (10)	0.61
Thoracic, n (%)	3	0 (0)	0.52	0 (0)	1
Lumbar, n (%)	13	5 (38.5)	0.67	4 (31)	0.32
Sex					
Female, n	12	5 (42)		4 (33)	
Male, n	14	3 (21)	0.67	1 (7)	0.14
Age, mean ± SD, years	45.2 ± 24 *	50.8 ± 14.8	0.60		
Age, mean ± SD, years	44.7 ± 16 **			56.4 ± 11.6	0.13
Resection					
GTR	22	7 (32)		5 (23)	
STR	4	1 (25)	1	0 (0)	0.55
Histology					
Schwannoma WHO I, n (%)	24	7 (29)		5 (21)	
Neurofibroma WHO I, n (%)	2	1 (50)	0.53	0 (0)	1
Sridhar grade					
Sridhar III, n (%)	8	1 (12.5)	0.36	0 (0)	0.15
Sridhar grade IV, n (%)	12	4 (33)	1	2 (17)	1
Sridhar grade V, n (%)	6	3 (50)	0.33	3 (50)	0.06
Diameter					
Transverse, mean ± SD, mm	43.5 ± 18.5 *	50.3 ± 7.6	0.33		
Transverse, mean ± SD, mm	41.8 ± 19.2 **			58.7 ± 15.3	0.08
Antero-posterior, mean ± SD, mm	33.9 ± 14.2 *	36.3 ± 6.3	0.65		
Antero-posterior, mean ± SD, mm	33.9 ± 12 **			36.3 ± 12	0.71

* Mean value for patients without immediate radicular deficit, ** mean value for patients without permanent radicular deficit, SD standard deviation, GTR gross total resection, STR subtotal resection.

**Table 6:** Correlation between baseline characteristics and postoperative immediate and persistent radicular deficit in the functional nerve roots.

Risk Factors/Variables	Population	Immediate Motor Deficit	*p* Value	Persistent Motor Deficit	*p* Value
Clinical presentation					
No preoperative motor deficit and/or sensory loss, n (%)	9	2 (22)		1 (11)	
Preoperative motor deficit and/or sensory loss, n (%)	5	3 (60)	0.26	1 (20)	1
No preoperative radicular pain, n (%)	6	0 (0)		0 (0)	
Preoperative radicular pain, n (%)	8	5 (63)	**0.03**	2 (25)	0.47
No preoperative myelopathy, n (%)	10	5 (50%)		2 (20)	
Preoperative myelopathy, n (%)	4	0 (0)	0.22	0 (0)	1
Medical history, mean ± SD, years	25.5 ± 20.7 *	21.2 ± 29.8	0.75		
Medical history, mean ± SD, years	27.8 ± 23.4 **			2 ± 0	0.15
No foraminal enlargement, n (%)	3	1 (33)		0 (0)	
Foraminal enlargement, n (%)	11	4 (36)	1	2 (18)	1
No extension in multiple foramen, n (%)	11	4 (36)		1 (9)	
Extension in multiple foramen, n (%)	3	1 (33)	1	1 (33)	0.39
Location					
Cervical, n	5	1 (20)		0 (0)	
Lumbar, n	9	4 (44)	0.58	2 (22)	0.50
Sex					
Female, n	7	4 (57)		1 (14)	
Male, n	7	1 (14)	0.26	1 (14)	1
Age, mean ± SD, years	47.6 ± 19.7 *	54.4 ± 9.3	0.45		
Age, mean ± SD, years	49 ± 16.8 **			56.5 ± 10.6	0.19
Resection					
GTR	12	5 (42)		2 (17)	
STR	4	0 (0)	0.10	0 (0)	1
Histology					
Schwannoma WHO I, n (%)	12	4 (33)		2 (17)	
Neurofibroma WHO I, n (%)	2	1 (50)	1	0 (0)	1
Tumor extension					
Sridhar grade III	3	0 (0)	0.25	0 (0)	1
Sridhar grade IV	7	3 (43)	1	0 (0)	0.46
Sridhar grade V	4	2 (50)	0.53	2 (50)	0.06
Diameter					
Transverse, mean ± SD, mm	38.6 ± 20.2 *	58.7 ± 9.3	0.06		
Transverse, mean ± SD, mm	41.9 ± 21 **			55.5 ± 10.6	0.39
Antero-posterior, mean ± SD, mm	30.6 ± 11.2 *	36.3 ± 7.7	0.32		
Antero-posterior, mean ± SD, mm	30.5 ± 10.3 **			39.5 ± 7.8	0.26

* Mean value for patients without immediate radicular deficit, ** mean value for patients without permanent radicular deficit, SD standard deviation, GTR gross total resection, STR subtotal resection.

## Data Availability

Not applicable.
